# Case Report: Left ventricular assist device implantation combined with cryoballoon ablation for ventricular tachycardia

**DOI:** 10.3389/fsurg.2024.1449007

**Published:** 2025-01-07

**Authors:** Shuang Zhang, Jianming Li, Changming Tan, Mingxian Chen, Lin Hu, Hanze Tang, Liyi Liao, Xuping Li

**Affiliations:** ^1^ Department of Cardiovascular Medicine, The Second Xiangya Hospital, Central South University, Changsha, Hunan, China; ^2^ Nuclear Medicine Department, Tianjin Medical University Clinical Cardiovascular Institute, TEDA International Cardiovascular Hospital, Tianjin, China; ^3^ Department of Cardiovascular Surgery, The Second Xiangya Hospital, Central South University, Changsha, Hunan, China

**Keywords:** left ventricular assist device implantation, cryoballoon ablation, ventricular tachycardia, ventricular arrhythmias, hybrid procedures

## Abstract

We report a case of a patient with dilated cardiomyopathy who experienced recurrent ventricular tachycardia (VT) and multiple defibrillations following CRT-D implantation. Due to worsening cardiac function, the patient required surgical implantation of a left ventricular assist device (LVAD) as a bridge to heart transplantation. During the procedure, we used the Ensite three-dimensional mapping system to perform activation and substrate mapping of the VT targets, followed by endocardial and epicardial cryoballoon ablation of clinical VT. Subsequently, during LVAD implantation, dual cryoballoon ablation was applied around the surgical incision site to prevent VT associated with the surgical wound and the implanted device. At the 1-year follow-up, the patient had no recurrence of the original clinical VT and no new ventricular arrhythmias were observed.

## Introduction

A 40-year-old male patient was admitted for the first time on January 29, 2021, 12 days after cardiopulmonary resuscitation, due to recurrent chest tightness and shortness of breath over the past 10 years. Cardiac Magnetic Resonance Imaging and echocardiography revealed global cardiac enlargement and significantly reduced ejection fraction (Left Atrium systolic dimension (LAS) 52 mm, Left ventricularend diastolic dimension (LVD) 85 mm, Right Atrium systolic dimension (RAS) 50 mm, Right Ventricular diastolic dimension (RVD) 42 mm, Ejection Fraction (EF) 22%). He was diagnosed with non-ischemic dilated cardiomyopathy. A 24-h Holter monitor performed after admission indicated 3,455 episodes of multifocal premature ventricular contractions and three episodes of nonsustained ventricular tachycardia (VT). Following comprehensive evaluation, cardiac resynchronization therapy-defibrillator (CRT-D) implant was implanted on February 18, 2021.

Monitoring captured episodes of VT, identified as monomorphic VT, likely scar-related arrhythmia. Due to the absence of chest leads on the monitor, only limb lead electrocardiograms were available for analysis. Based on the electrocardiogram (ECG), the VT was preliminarily determined to originate from the lower ventricle. The biphasic waveform in lead I suggested that the arrhythmia might have originated from the septal region. The VT episodes occurred spontaneously and were terminated by the CRT-D device. Given the patient's severely impaired cardiac function, with an EF of only 22%, and the accompanying hemodynamic instability, initial management prioritized medication and CRT-D implantation over VT ablation.

Despite oral amiodarone and metoprolol sustained-release tablets, the patient continued to experience VT episodes, with 30 defibrillator discharges between April 4, 2022, and April 25, 2023. After multiple hospital evaluations, heart transplantation was considered, and the patient was readmitted on April 25, 2023, for planned left ventricular assist device (LVAD) implantation.Considering the patient's recurrent VT episodes despite medication, ventricular tachycardia ablation was performed under pre-visualized guidance simultaneously with the LVAD implantation.

**Figure 1:**
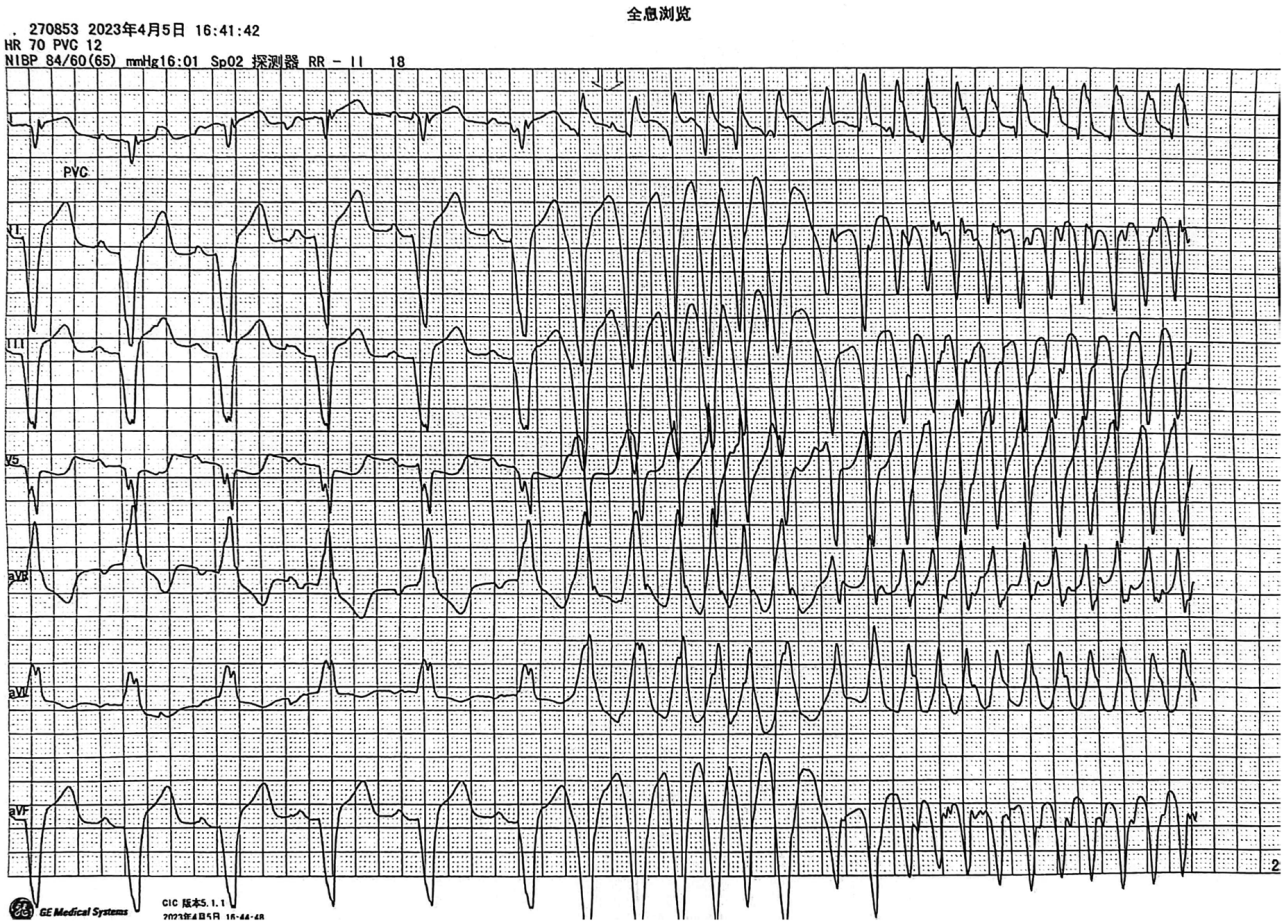
Ventricular tachycardia episode captured on the patient's cardiac monitor.

**Figure 2:**
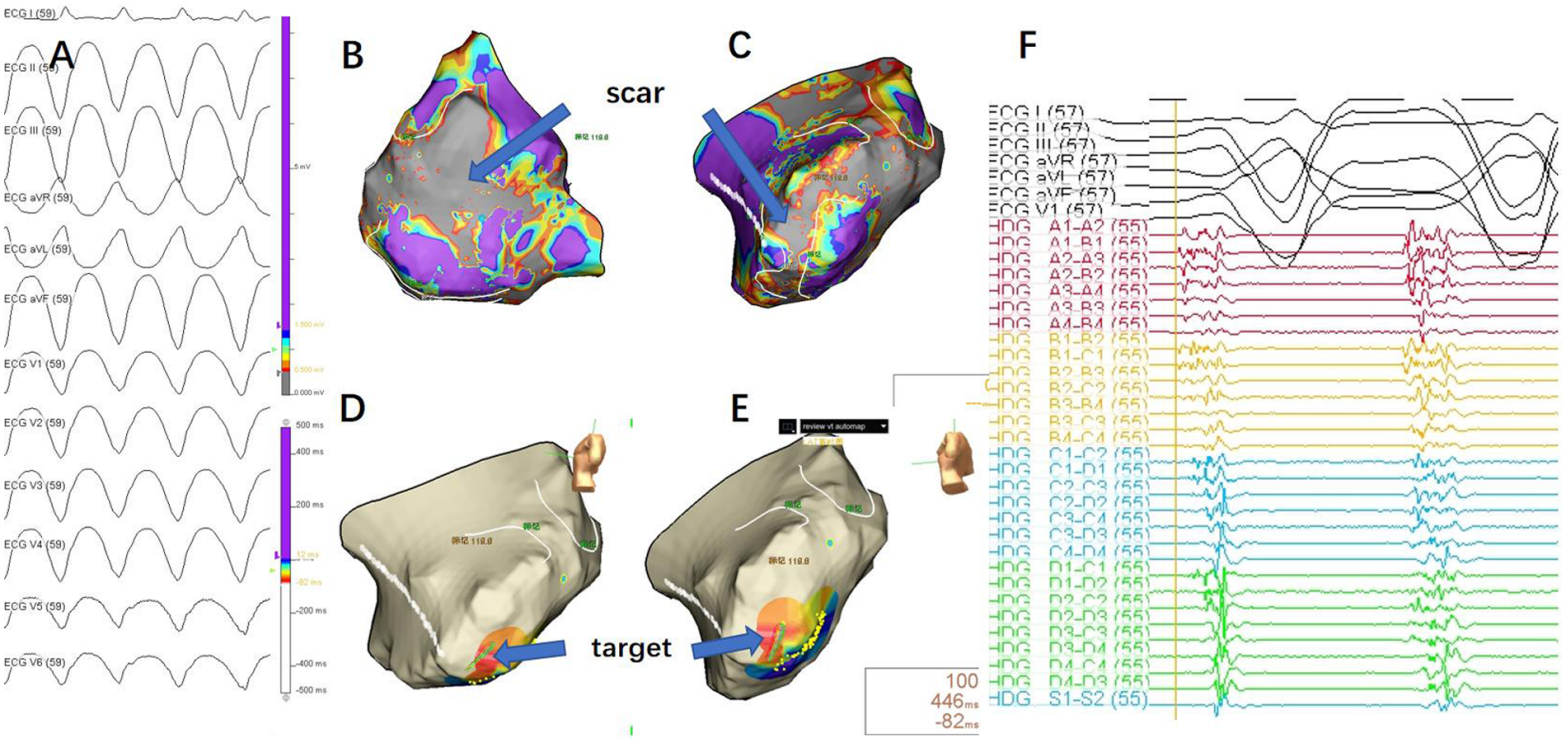
**(A)** 12-lead ECG during ventricular tachycardia (VT) episode.Due to the requirement for an anterior thoracotomy, the precordial leads were placed on the iliac crest. As a result, the precordial lead reference differed from the VT morphology typically observed during the patient's daily episodes, but the limb lead morphology was consistent with the patient's daily VT episodes. **(B)** Epicardial substrate map during sinus rhythm (posterior-anterior view), with the scar area shown in gray, indicating no electrical activity. **(C)** Epicardial substrate map during sinus rhythm (left lateral view), with the scar area shown in gray, indicating no electrical activity. **(D, E)** Mapping during VT, with the earliest activation point indicated by the blue arrow. **(F)** Mapping target potential, showing fragmented waves in high-density mapping, significantly preceding the QRS complex in the surface ECG.

Epicardial mapping (Ensite, St. Jude Medical) was performed through a subxiphoid approach without cardiopulmonary bypass. During sinus rhythm, a large low-voltage area was observed in the left ventricle (Figures 2B, C). The induced VT (Figure 2A) was similar to the patient's symptomatic episodes (Figure 1). Due to the non-sustained nature of the VT, short bursts of ventricular stimulation were used to induce VT for mapping. The earliest activation points during VT coincided with the low-voltage scar areas observed during sinus rhythm, with significant fragmented potentials preceding the surface QRS complex (Figures 2D, E, F).

**Figure 3:**
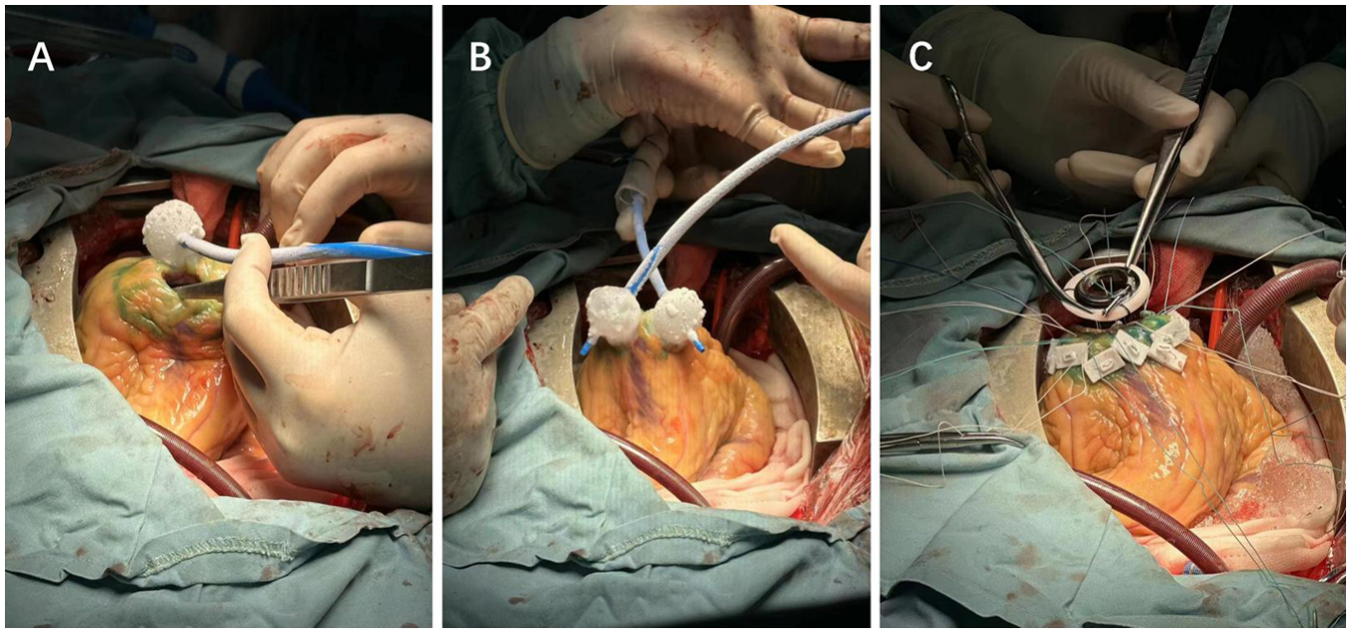
**(A)** Cryoablation of scar-related ventricular tachycardia. **(B)** Alternating ablation of the LVAD device incision site using dual cryoballoons. **(C)** Suturing after the completion of LVAD implantation and ablation.

Considering the need for LVAD implantation and to prevent scar-related reentrant arrhythmias at the inflow cannula and left ventricular apex anastomosis site, ablation was necessary at the incision site. Given the limited treatment area per application with radiofrequency ablation, cryoballoon ablation (Arctic Front Advance Cryoablation, Medtronic Inc) was chosen to shorten the procedure time and ensure safety. Guided by three-dimensional mapping, the target areas were delineated, and scar-related VAs were ablated using cryoballoon technology (Figure 3A), including the left ventricular apex inflow cannula anastomosis site (Figure 3B).

To further reduce operative time, dual cryoballoons were used alternately and continuously to ablate the incision area (Figure 3B). This combined strategy of three-dimensional mapping and cryoablation also allowed verification of the effectiveness of cryoablation.

## Outcom and follow up

The patient remained in sinus rhythm after discontinuing cardiopulmonary bypass and closing the chest cavity. Given the patient's heart failure, they have been consistently taking sacubitril/valsartan and metoprolol extended-release tablets to manage the condition.

After the surgery in May 2023, the patient has been receiving phone follow-ups every 3 months. If the patient experiences a shock sensation, they are instructed to seek medical attention immediately. If no shock sensation occurs during the phone follow-up, CRTD programming is performed every six months. During the follow-up period, the programming device indicated no episodes of ventricular tachycardia. In March 2024, the patient was readmitted due to a lung infection, and the CRTD programming during this hospitalization also showed no ventricular arrhythmias. The next CRTD programming is scheduled for September. The patient is currently still awaiting a heart transplant.

## Discussion

The LVAD plays an increasingly important role in the treatment of patients with end-stage heart failure with reduced ejection fraction. While LVADs effectively bridge patients to heart transplantation and improve survival rates, ventricular arrhythmias (VAs) are common in patients with LVADs (1–4), and conservative treatments are often ineffective.

VAs may be related to the underlying heart disease or the LVAD implantation itself. The LVAD inflow cannula is anastomosed to the left ventricular apex, and extracorporeal circulation is established through the right atrial appendage. If mitral and tricuspid valve repairs are required, an incision in the anterior atrial wall is made. These sites are potential substrates for scar-related arrhythmias (4). Additionally, acute left ventricular underfilling or high pump speeds may cause the LVAD inflow cannula to contact the ventricular wall, leading to arrhythmias (5). At the cellular level, VT is associated with the β-adrenergic system and calcium regulation (5). While the latter types of VTs can be improved by adjusting LVAD parameters and oral medications, scar-related arrhythmias are typically not resolved through these measures.

Some patients may have an implantable cardioverter-defibrillator (ICD) or CRT-D before LVAD implantation. Studies have shown that preoperative VAs are predictors of post-LVAD VA occurrences (2, 6). Post-LVAD ICD implantation effectively reduces postoperative VT episodes (7), with CRT-D implantation having similar effects to ICDs (8). However, ICD or CRT-D discharges are associated with frequent rehospitalizations, posing additional risks and complicating patient management.

Catheter ablation is an effective treatment for refractory VAs, reducing ICD shocks and patient discomfort. Most VAs in LVAD patients are monomorphic and sustained, often related to pre-existing scar tissue, with fewer related to the apical cannula (9–11). To mitigate or eliminate these arrhythmias, hybrid surgical and catheter ablation techniques are promising (4). Studies have shown that catheter ablation is effective (9, 11), and localized cryoablation during LVAD implantation can also reduce postoperative VA episodes (12).

In this case report, the patient experienced VT before surgery. A three-dimensional anatomical reconstruction was performed before LVAD implantation, and the mapping system identified scar-related reentry. To reduce LVAD-related VA occurrences, intraoperative cryoablation was applied to both the existing scar-related reentry substrate and the apical cannula site.

## Data Availability

The original contributions presented in the study are included in the article/Supplementary Material, further inquiries can be directed to the corresponding author.
